# Specialists

**Published:** 1920-06

**Authors:** Julio Edelman


### SPECIALISTS

BY JULIO EDELMAN, D.D.S.

We are from time to time consulted by young men on the verge of graduation and by graduates of recent standing, concerning their desire to enter the practice of dentistry as full-fledged specialists. There are to be found among dental students few who ever attain a sufficient degree of proficiency in all departments of dentistry; and, as a matter of fact, the parallel of these conditions will also be found to exist among the more mature practitioners, even after years of clinical experience. With the increase in knowledge in every branch of dentistry, by a process of rapid-fire evolution, the scope in each professional department has become so extensive and the respective technique of restorative procedures so exacting, that it is no wonder that thoughts of uncertainty and doubt find their way into the minds of prospective practitioners. To engage in the practice of all branches of dentistry and to render honest services to patients requiring a diversity of operations is a proven impossibility. In the face of average human limitations, to excel in every division and subdivision of the science and art of dentistry approaches in its complexity the unloosing of the Gordian knot. Of the specialist, however, the public demands and should receive expert advice and service; and because his field is restricted, he should stand head and shoulders above everyone else in the profession who does not build a barrier between the kind of dental services that a specialist renders and the kind from which he has divorced himself. The ability of the specialist to render services commensurate with his self-assumed position of expert and leader in a limited territory, rests upon a foundation made up of several imperative requirements. A dental practitioner's ambition to specialize is not infrequently the expression of a wish, rather than of a conviction of scientific and technical excellence. The term specialist should be a thoroughly deserved designation, and in its unuttered meaning should be embodied the thought of years of work in a given field,—of years of preparation to render professional services upon a plane of a maximum of physiologic and not necessarily of pecuniary returns. In the average general practice only a limited number of cases of any given kind are observable in one year's time, and even though reinforced by special courses and individual concentration of effort, we dare say that ten years of general practice is the minimum requirement before taking one's place in the arena of professional accomplishment as a well-rounded specialist. Using the so-called pyorrhea specialist as an example of the opinions we are endeavoring to clearly record, it is a prevailing belief that the ability to thoroughly remove hard and soft deposits from the surfaces of teeth is the alpha and omega of ultimate success. This empirical conception of the treatment of the investing tissues is leading to disastrous results. What is the impression gathered from a post-graduate course on pyorrhea? From such courses, for example, as have been frequently given in the past, and through the acquiescence of the dental profession will probably continue to bob up in the future? All patients are given the identical treatment; no efforts are made to eliminate the factors originally responsible for the infection and subsequent destruction of the investing tissues; no consideration is given to the systemic background, different in practically every case. The requirements to render service in this field are not measured by the ability to scrape and polish hard tooth surfaces, but by a thorough familiarity with every phase of the subject, technical and scientific. He should be familiar with the literature of the subject and should keep abreast of the time by consistently reading all articles bearing on his subject which may appear in the standard dental magazines; he should be professionally broad-minded to profit by the experience of brother practitioners in the same field; he should be a competent diagnostician, and inasmuch as skill in diagnosis is the reflection of a trained sense of observation and of the ability to draw conclusions upon a basis of scientifically established facts, *ipso facto,* the specialist should be an embodiment of sincere attachment to all such scientific subjects as may assist him in the noble task of adding happiness to the lives of his patients.

Could all this be accomplished upon a foundation of one or two years in general practice with perhaps no effort, after graduation, to broaden the fundamental knowledge acquired during the period of studentship? ''And what's impossible can't be, and never, never comes to pass.''—*Pacific Dental Gazette.*

